# Nicotinamide Mononucleotide and Nicotinamide Riboside Improve Dyslipidemia and Fatty Liver but Promote Atherosclerosis in Apolipoprotein E Knockout Mice

**DOI:** 10.3390/ph18030281

**Published:** 2025-02-20

**Authors:** Pin Wang, Jia-Xin Li, Yuan-Yuan Kong, Si-Li Zheng, Chao-Yu Miao

**Affiliations:** Department of Pharmacology, Second Military Medical University/Naval Medical University, Shanghai 200433, China; wpin2018@163.com (P.W.); ljxin@smmu.edu.cn (J.-X.L.); yaolikyy@163.com (Y.-Y.K.); slzheng@smmu.edu.cn (S.-L.Z.)

**Keywords:** nicotinamide mononucleotide, nicotinamide riboside, ApoE knockout mice, fatty liver, atherosclerosis

## Abstract

**Background:** Nicotinamide mononucleotide (NMN) and nicotinamide riboside (NR) are intermediary products in NAD+ metabolism. NMN and NR supplementation can elevate NAD+ levels in tissues, addressing health issues associated with aging and obesity. However, the impact of NMN and NR on atherosclerosis remains incompletely elucidated. **Methods:** C57BL/6J and Apolipoprotein E knockout (ApoE^−/−^) mice were used to explore the impact of NMN and NR supplementation on serum lipids, fatty liver, and atherosclerosis. Additionally, various suppliers, administration protocols, and doses on ApoE^−/−^ mice were investigated. **Results:** The intragastric administration of NMN (300 mg/kg) and NR (230 mg/kg) reduced body weight, serum lipids, and fatty liver but aggravated atherosclerosis in ApoE^−/−^ mice after 4 months of administration with different suppliers. Atherosclerosis also deteriorated after 2 months of different NMN administration protocols (intragastric and water administration) in ApoE^−/−^ mice with existing plaques. The effects of NMN were dose-dependent, and doses around 100 mg/kg had little harmful effects on atherosclerosis. **Conclusions:** NMN and NR improve dyslipidemia and fatty liver but promote atherosclerosis in ApoE^−/−^ mice. These findings emphasize the safe dosage for the clinical trials of NMN.

## 1. Introduction

Nicotinamide adenine dinucleotide (NAD+) is an essential pyridine compound for cellular bioenergetics and metabolism, such as DNA damage repair, epigenetic modifications, cellular autophagy, and aging [1,2]. In mammalian cells, NAD+ is mainly synthesized through the salvage pathway [3]. Nicotinamide mononucleotide (NMN) is synthesized by the mammalian NAD+ biosynthetic rate-limiting enzyme nicotinamide phosphoribosyltransferase (Nampt) from nicotinamide (NAM) and can also be produced from nicotinamide riboside (NR) through a phosphorylation reaction mediated by nicotinamide riboside kinases (NRKs) and further catalyzed by nicotinamide mononucleotide adenylyltransferases (NMNATs) to convert to NAD+ [4,5]. Numerous animal studies have shown that regular supplementation of NMN can effectively enhance the biosynthesis of NAD+ in various peripheral tissues, such as the liver, adipose, heart, aorta, and brain tissue [6,7,8]. NMN supplementation has beneficial effects on various key physiological functions in experimental animals, demonstrating significant therapeutic potential in the regulation of glucose and lipid metabolism, neuronal protection, and anti-aging effects [9,10].

Atherosclerosis, a chronic inflammatory disease, is distinguished by the accumulation of lipids that predominantly afflicts large and medium-sized arteries, serving as the fundamental pathological alteration in major cardiovascular diseases, such as myocardial infarction and stroke [11]. Dyslipidemia, endothelial dysfunction, and obesity can promote the formation of atherosclerotic plaques [12]. In recent years, more and more clinical studies have revealed the association between fatty liver disease and atherosclerosis. Excessive lipid accumulation in the liver can stimulate inflammation response, enhance insulin resistance, and impair the biological processes of hepatic cholesterol transport, which leads to a greater risk of vascular disease [13,14]. Due to the pivotal role of disrupted lipid metabolism in the pathogenesis of atherosclerosis, lipid-lowering drugs have been utilized as the cornerstone of clinical treatment for it, including statins, cholesterol absorption inhibitors, and proprotein convertase subtilisin kexin/type9 (PCSK9) inhibitor [15,16]. Although current studies have also shown the beneficial effects of NMN and NR in improving impaired glucose tolerance and dyslipidemia induced by high-fat diet (HFD) in mice [4,17], their impacts on atherosclerosis are still controversial.

On the one hand, exogenous supplementation of NMN and NR enhances the ability of NAD+ biosynthesis through a series of enzymatic reactions and has the effect of protecting atherosclerosis. In vitro, NMN and NR regulate the content of NAD+ in endothelial cells through CD73 pathways, inhibiting endothelial inflammation and improving endothelial dilation function. In vivo, NMN restores vascular SIRT1 activity in aging mice by reducing oxidative stress, rescues carotid artery endothelium-dependent dilatation damage, and protects aging-related arterial dysfunction [18]. miRNA expression profiles in the aorta of some aging mice are reversed by NMN treatment, thereby improving the bioavailability of nitric oxide and inhibiting atherosclerosis [19]. On the other hand, NAD+-dependent biochemical metabolic reactions involve complex interactions between various pathways. Some studies have yielded conflicting results regarding the role of the Nampt-NAD axis in atherosclerosis. In patients with carotid atherosclerosis, serum Nampt levels are significantly increased [20]. The inhibition of Nampt expression can reduce CXCL1-mediated neutrophil activity to alleviate the level of inflammation in atherosclerotic plaques [21]. Nampt-specific knockdown can promote reverse cholesterol transport and attenuate atherosclerosis in Apolipoprotein E knockout (ApoE^−/−^) mice [22]. Our previous studies also showed that global overexpression of Nampt significantly increased the levels of inflammation and exacerbated atherosclerosis [23].

It is worth noting that guidelines around the world emphasize the long-term, personalized, and preventive nature of atherosclerosis drug therapy [24]. The pharmacological effects of NMN and NR in the human body are extremely complex, and the knowledge of this complex is still limited. Furthermore, the dosage of NMN and NR has been largely overlooked in current research. High doses of NMN supplementation far exceed the body’s requirement for NAD+ equivalent and may potentially pose a metabolic burden. Currently, NMN is commonly utilized as a nutraceutical in various populations at doses ranging from 50 to 150 mg/d, while NR can reach 1 g/d [10]. It is crucial to thoroughly evaluate the potential side effects of prolonged administration therapy on atherosclerosis. In addition, the metabolism of NMN exhibits tissue specificity, and NMN supplements may not be suitable for all individuals. People are more susceptible to atherosclerosis, especially the elderly, who are the main population for NMN supplementation and require special attention to its safety. Therefore, this study aimed to elucidate the long-term effects of NMN and NR supplementation on serum lipids, liver condition, and atherosclerosis in ApoE^−/−^ mice with HFD, and different administration protocols and dosages were also firstly investigated so as to provide a basis for relatively safe nutraceutical applications and clinical trials.

## 2. Results

### 2.1. NMN and NR Improve Dyslipidemia and Fatty Liver in C57BL/6J Mice Fed HFD

In the experiment, we used various products from different suppliers for comparative study. NMN1 was provided by SYNCOZYMES Co., Ltd. (Shanghai, China). NR1 was provided by Natural Field Bio-Technique Co., Ltd. (Xi’an, China). NMN2 and NR2 were provided by EffePharm Co., Ltd. (Shanghai, China), and NMN3 was provided by Bontac Bio-engineering Co., Ltd. (Shenzhen, China).

**Figure 1:**
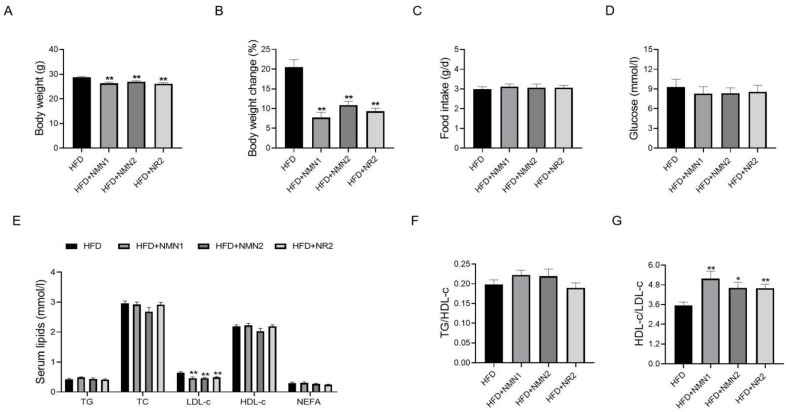
Effects of NMN1 (300 mg/kg), NMN2 (300 mg/kg), and NR2 (230 mg/kg) on body weight, food intake, glucose, and serum lipids in C57BL/6J mice with HFD for 1 month. (**A**) Body weight (*n* = 10), (**B**) body weight change (*n* = 10), (**C**) food intake (*n* = 10), (**D**) glucose (*n* = 10), (**E**) serum lipids (*n* = 10), (**F**,**G**) TG to HDL-c, (**F**) and the LDL-c to HDL-c (**G**) ratio (*n* = 10) at 1 month after treatment. Data represent the mean ± SEM, * *p* < 0.05, ** *p* < 0.01 vs. HFD group. NMN: nicotinamide mononucleotide, NR: nicotinamide riboside, TG: triglyceride, TC: total cholesterol, LDL-c: low-density lipoprotein cholesterol, HDL-c: high-density lipoprotein cholesterol, NEFA: non-esterified fatty acid.

To investigate the effects of NMN and NR on the body weight, food intake, glucose, and serum lipids in C57BL/6J mice fed HFD for 1 month, the three administration groups were given NMN1 (300 mg/kg), NMN2 (300 mg/kg), and NR2 (230 mg/kg) by intragastric administration daily during HFD period, while the control group was given water intragastrically. Body weight and body weight change were significantly decreased in the three administration groups (Figure 1A,B, *p* < 0.05), but there were no significant changes in the three groups in terms of food intake and glucose at 1 month (Figure 1C,D). Serum lipids, including triglyceride (TG), low-density lipoprotein cholesterol (LDL-c), high-density lipoprotein cholesterol (HDL-c), and non-esterified fatty acid (NEFA) were also examined. LDL-c levels were lower in mice given NMN or NR for 1 month than in controls, but TG, TC, HDL-c, and NEFA levels were not statistically different (Figure 1E). In addition, compared with the control group, the LDL-c/HDL-c ratio decreased, but the TG/HDL-c ratio did not change significantly in the three treatment groups (Figure 1F,G).

**Figure 2:**
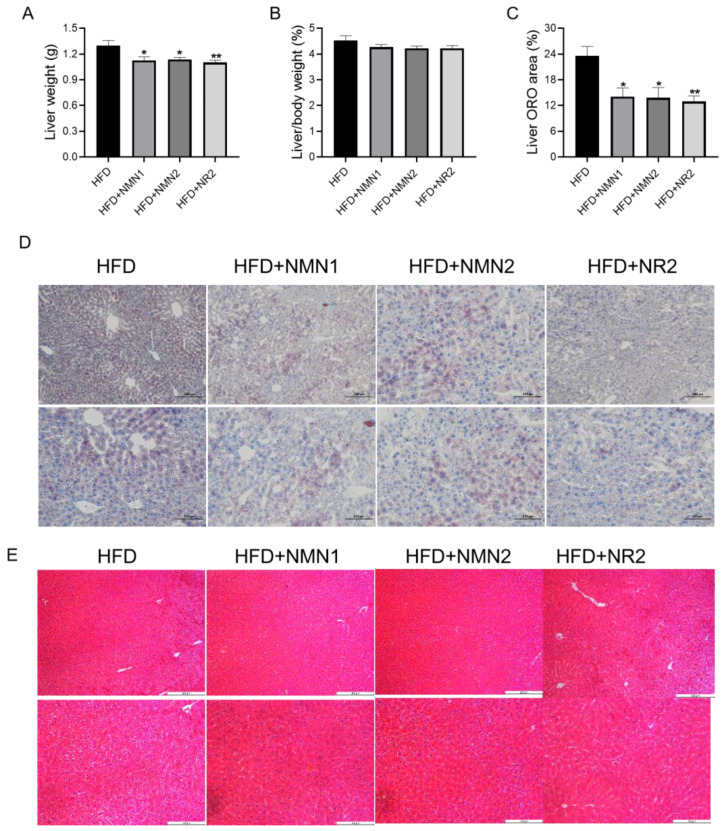
Effects of NMN1 (300 mg/kg), NMN2 (300 mg/kg), and NR2 (230 mg/kg) on liver in C57BL/6J mice with HFD for 1 month. (**A**) Liver weight (*n* = 10), (**B**) liver-to-body-weight ratio (*n* = 10), (**C**) liver ORO areas (*n* = 10), (**D**,**E**) the representative images of Oil red O (**D**) and HE (**E**) staining on the liver at 1 month after treatment. Data represent the mean ± SEM, * *p* < 0.05, ** *p* < 0.01 vs. HFD group. 100× or 200×. NMN: nicotinamide mononucleotide, NR: nicotinamide riboside.

Our previous study showed that the addition of NR to the diet attenuated HFD-induced hepatic steatosis [18]. We further examined the liver in the treated mice. Compared with the control group, the liver weight of mice significantly decreased after 1 month of treatment (Figure 2A, *p* < 0.05), and the liver-to-body weight ratio showed a trend to decrease (Figure 2B). The supplement of NMN and NR also reduced liver Oil red O staining areas in the mice (Figure 2C,D). Meanwhile, HE staining showed that NMN and NR supplementation alleviated hepatic injuries and fatty degeneration (Figure 2E). These results indicate that supplementation with NMN and NR improves dyslipidemia and fatty liver in C57BL/6J mice.

### 2.2. NMN and NR Improve Dyslipidemia and Fatty Liver in ApoE^−/−^ Mice Fed HFD

**Figure 3:**
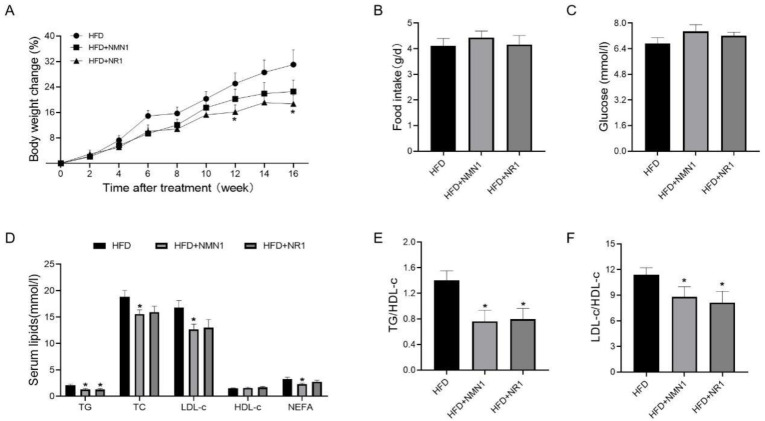
Effects of NMN1 (300 mg/kg) and NR1 (230 mg/kg) on body weight, food intake, glucose, and serum lipids in ApoE^−/−^ mice with HFD for 4 months. (**A**) Body weight change (*n* = 9–10), (**B**) food intake (*n* = 8), (**C**) glucose (*n* = 10), (**D**) serum lipids (*n* = 9–10), (**E**,**F**) TG to HDL-c, (**E**) and LDL-c to HDL-c (**F**) ratio (*n* = 9–10) at 4 months after treatment. Data represent the mean ± SEM, * *p* < 0.05 vs. HFD group. NMN: nicotinamide mononucleotide, NR: nicotinamide riboside, TG: triglyceride, TC: total cholesterol, LDL-c: low-density lipoprotein cholesterol, HDL-c: high-density lipoprotein cholesterol, NEFA: non-esterified fatty acid.

**Figure 4:**
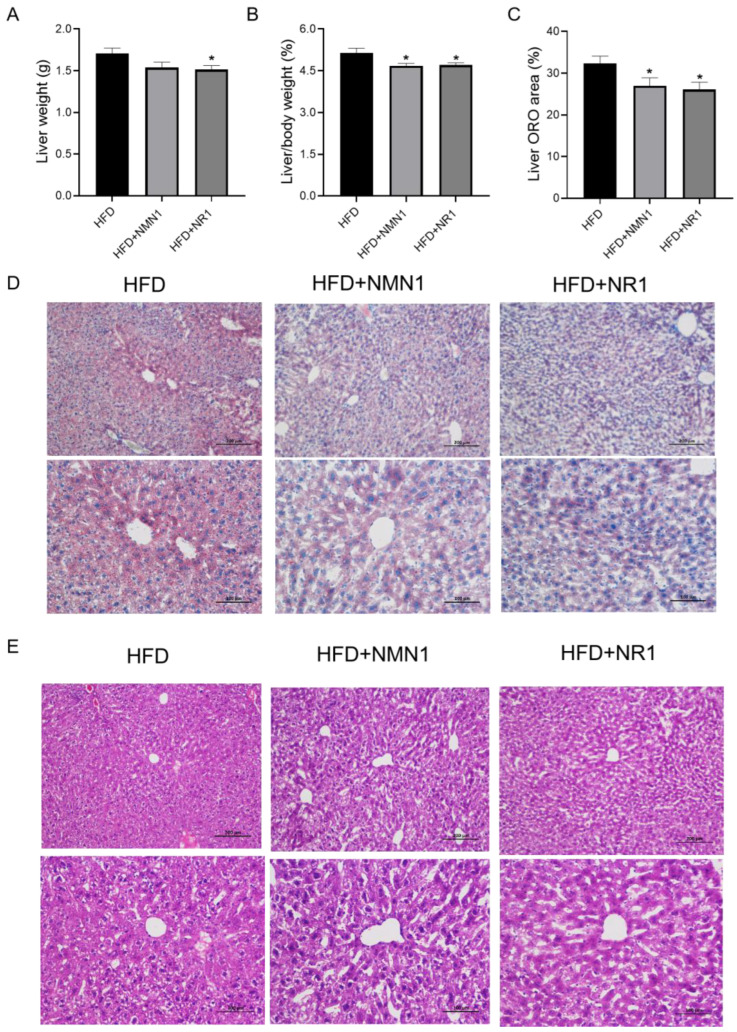
Effects of NMN1 (300 mg/kg) and NR1 (230 mg/kg) on the liver in ApoE^−/−^ mice with HFD for 4 months. (**A**) Liver weight (*n* = 9–10), (**B**) liver-to-body-weight ratio (*n* = 9–10), (**C**) liver ORO area (*n* = 8), (**D**,**E**) the representative images of Oil red O (**D**) and HE (**E**) staining on the liver at 4 months after treatment. Data represent the mean ± SEM, * *p* < 0.05 vs. HFD group. 100× or 200×. NMN: nicotinamide mononucleotide, NR: nicotinamide riboside.

We assessed the effects of NMN and NR treatment in ApoE^−/−^ mice fed HFD for 4 months by intragastric administration of NMN1 (300 mg/kg) and NR1 (230 mg/kg) to the treated groups of ApoE^−/−^ mice for 4 months, with the control group given water intragastrically. Body weight gain tended to reduce in NMN- and NR-treated groups, with a significant reduction in NR1 treatment at 12 and 16 weeks (Figure 3A, *p* < 0.05), and there were no significant changes in food intake and glucose in treated groups (Figure 3B,C). Serum lipids, including TG, TC, LDL-C, and NEFA, appeared reduced, with more efficiency by NMN1 than NR1 (Figure 3D). Both TG/HDL-c and LDL-c/HDL-c ratios were reduced by the treatments. Fatty liver was also improved by the treatments, with reductions in liver weight (Figure 4A,B), lipid accumulation (Figure 4C,D), and injury (Figure 4E).

### 2.3. NMN (300 mg/kg) and NR (230 mg/kg) Promote Atherosclerosis in ApoE^−/−^ Mice Fed HFD

**Figure 5:**
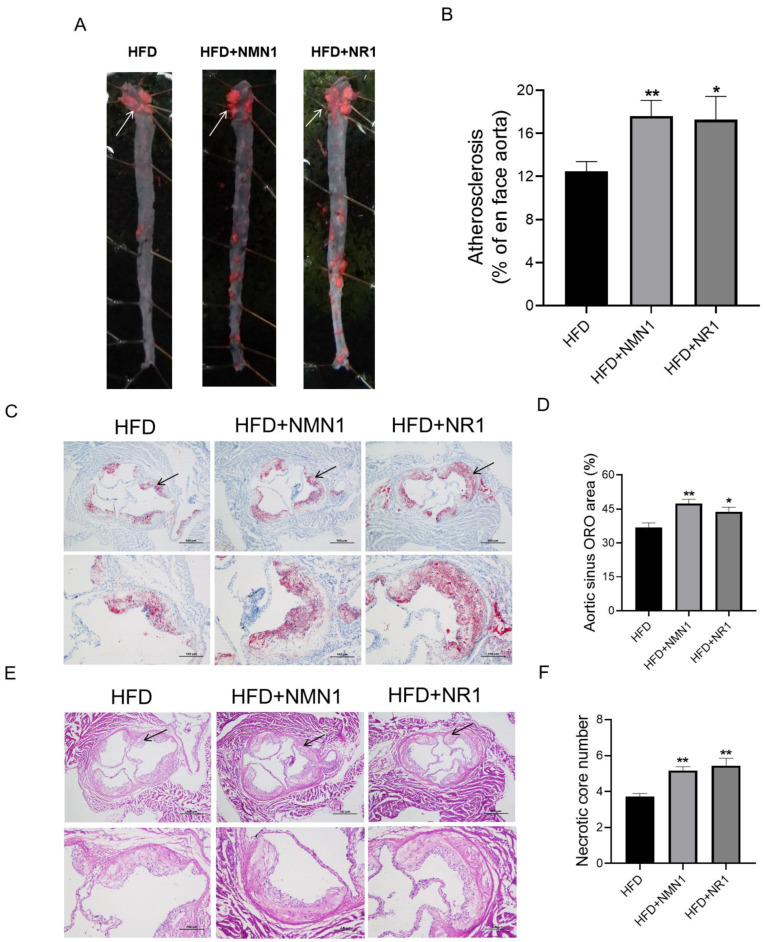
Effects of NMN1 (300 mg/kg) and NR1 (230 mg/kg) on atherosclerosis in ApoE^−/−^ mice with HFD for 4 months. (**A**,**B**) The representative images of Oil red O staining on the aorta (**A**) and the statistical results (**B**) (*n* = 10–18), (**C**,**D**) the representative images of Oil red O staining on the aortic root (**C**) and the statistical results (**D**) (*n* = 10–18), (**E**,**F**) the representative images of HE on the aortic root, (**E**) and the statistical results (**F**) (*n* = 10–18) at 4 months after treatment. Data represent the mean ± SEM, * *p* < 0.05, ** *p* < 0.01 vs. HFD group. The white arrows point to stained atherosclerotic plaques (**A**). The black arrows point to lipid deposits (**C**) and necrotic areas (**E**) at the root of the aorta. 40× or 100×. NMN: nicotinamide mononucleotide, NR: nicotinamide riboside.

The above-treated ApoE^−/−^ mice were used for the examination of atherosclerosis. Both NMN1 and NR1 treatments aggravated atherosclerosis, with atherosclerotic plaque increases in aorta (Figure 5A,B) and aortic sinus (Figure 5C,D), as well as necrotic core number increases in aortic sinus (Figure 5E,F).

**Figure 6:**
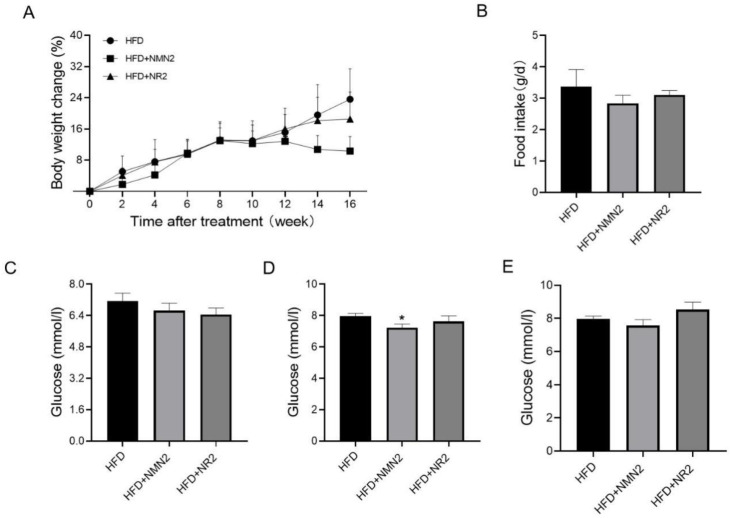
Effects of NMN2 (300 mg/kg) and NR2 (230 mg/kg) on body weight, food intake, and glucose in ApoE^−/−^ mice with HFD for 4 months. (**A**) Body weight change (*n* = 8), (**B**) food intake (*n* = 8), (**C**–**E**) glucose at 2, 3 and 4 months (*n* = 8) after treatment. Data represent the mean ± SEM, * *p* < 0.05 vs. HFD group. NMN: nicotinamide mononucleotide, NR: nicotinamide riboside.

**Figure 7:**
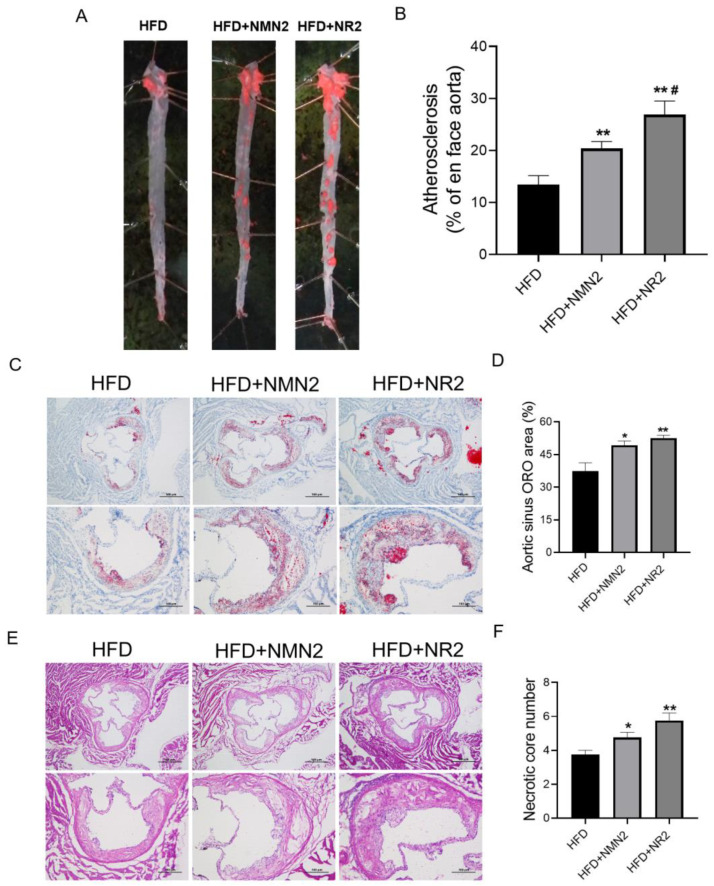
Effects of NMN2 (300 mg/kg) and NR2 (230 mg/kg) on atherosclerosis in ApoE^−/−^ mice with HFD for 4 months. (**A**,**B**)The representative images of Oil red O staining on the aorta (**A**) and the statistical results (**B**) (*n* = 8), (**C**,**D**) the representative images of Oil red O staining on the aortic root (**C**) and the statistical results (**D**) (*n* = 8), (**E**,**F**) the representative images of HE on the aortic root (**E**) and the statistical results (**F**) (*n* = 8) at 4 months after treatment. Data represent the mean ± SEM, * *p* < 0.05, ** *p* < 0.01 vs. HFD group, ^#^ *p* < 0.05 vs. HFD + NMN2 group. 40× or 100×. NMN: nicotinamide mononucleotide, NR: nicotinamide riboside.

In order to further clarify these aggravation effects on atherosclerosis, NMN2 and NR2 were tested. Although there were no obvious changes in body weight, food intake, and blood glucose at 2, 3, and 4 months after treatment (Figure 6A–E), the results definitely showed that 4 months of both NMN2 and NR2 treatments also aggravated atherosclerosis in HFD-fed ApoE^−/−^ mice (Figure 7A–F). It appeared that the aggravation effect on atherosclerosis was more severe in NR2 than in NMN 2 treatment (Figure 7A,B).

Other administration protocols and methods were used to further observe the effects of NMN on atherosclerosis.

### 2.4. More Protocols Confirm NMN Aggravation on Atherosclerosis in ApoE^−/−^ Mice Fed HFD

**Figure 8:**
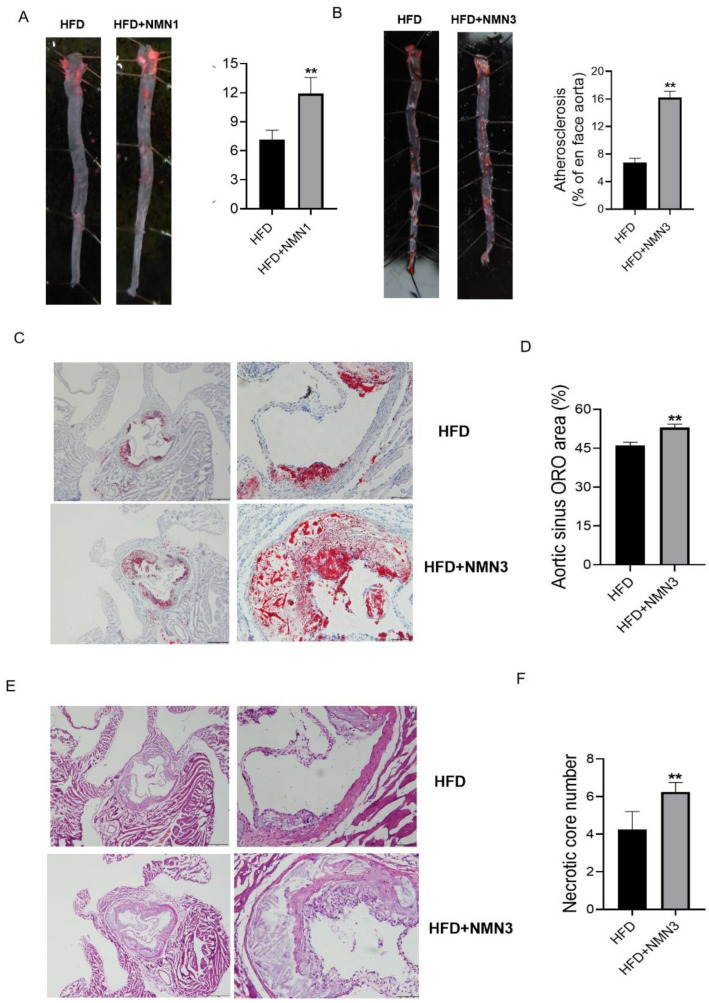
Effects of NMN1 (300 mg/kg, by gavage, 2 months) and NMN3 (300 mg/kg, by drinking water, 2 months) on atherosclerosis in ApoE^−/−^ mice with HFD for 4 months. (**A**) The representative images of Oil red O staining on the aorta and the statistical results with NMN1 (300 mg/kg, by gavage, 2 months) (*n* = 8–9), (**B**) the representative images of Oil red O staining on the aortic root and the statistical results with NMN3 (300 mg/kg, by drinking water, 2 months) (*n* = 4), (**C**,**D**)the representative images of Oil red O staining on the aortic root (**C**) and the statistical results (**D**) (*n* = 4) with NMN3 (300 mg/kg, by drinking water, 2 months), (**E**,**F**) the representative images of HE staining on the aortic root (**E**) and the statistical results (**F**) (*n* = 4) with NMN3 (300 mg/kg, by drinking water, 2 months). Data represent the mean ± SEM, ** *p* < 0.01 vs. HFD group. 40× or 100×. NMN: nicotinamide mononucleotide, NR: nicotinamide riboside.

Further verification was performed using other administration protocols in ApoE^−/−^ mice fed HFD for 4 months. Unlike before, NMN1 (300 mg/kg) was given intragastrically and daily for 2 months after 2 months of HFD-induced atherosclerosis formation in ApoE^−/−^ mice (4 months of HFD and 2 months of NMN1 intragastrically). This administration protocol of NMN1 also promoted atherosclerosis (Figure 8A, *p* < 0.05). Next, drinking water administration of NMN3 was used for the same duration and dose (4 months of HFD and 2 months of NMN3 by drinking water), and showed atherosclerotic plaque increases in aorta (Figure 8B, *p* < 0.05) and aortic sinus (Figure 8C,D, *p* < 0.05), as well as necrotic core number increases in aortic sinus (Figure 8E,F, *p* < 0.05). These results indicate that NMN, either by gavage or by drinking water, aggravates atherosclerosis when the mice have formed plaques on the aorta.

### 2.5. Good and Bad NMN Effects Decrease with Dose Reduction in ApoE^−/−^ Mice Fed HFD

**Figure 9:**
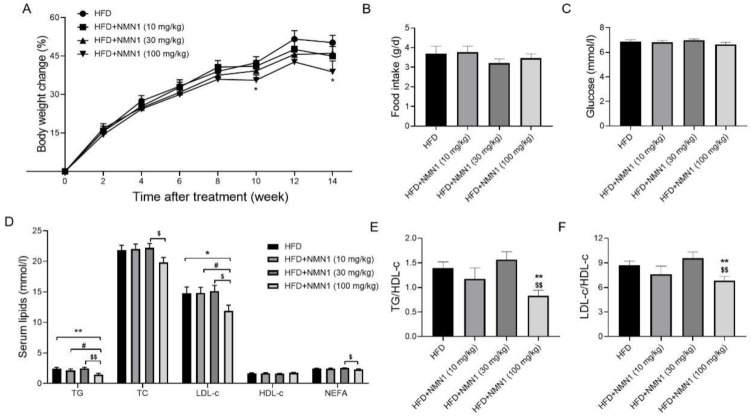
Effects of lower doses of NMN1 (10, 30, 100 mg/kg) on body weight, food intake, glucose, and serum lipids in ApoE^−/−^ mice with HFD for 14 weeks. (**A**) Body weight change (*n* = 11–12), (**B**) food intake (*n* = 11–12), (**C**) glucose (*n* = 11–12), (**D**) serum lipids (*n* = 11–12), (**E**,**F**) TG to HDL-c (**E**) and LDL-c to HDL-c (**F**) ratio (*n* = 11–12) at 14 weeks after treatment. Data represent the mean ± SEM, * *p* < 0.05, ** *p* < 0.01 vs. HFD group; ^#^ *p* < 0.05 vs. HFD+NMN1 (10 mg/kg) group; ^$^ *p* < 0.05, ^$$^ *p* < 0.01 vs. HFD + NMN1 (30 mg/kg) group. NMN: nicotinamide mononucleotide, NR: nicotinamide riboside, TG: triglyceride, TC: total cholesterol, LDL-c: low-density lipoprotein cholesterol, HDL-c: high-density lipoprotein cholesterol, NEFA: non-esterified fatty acid.

**Figure 10:**
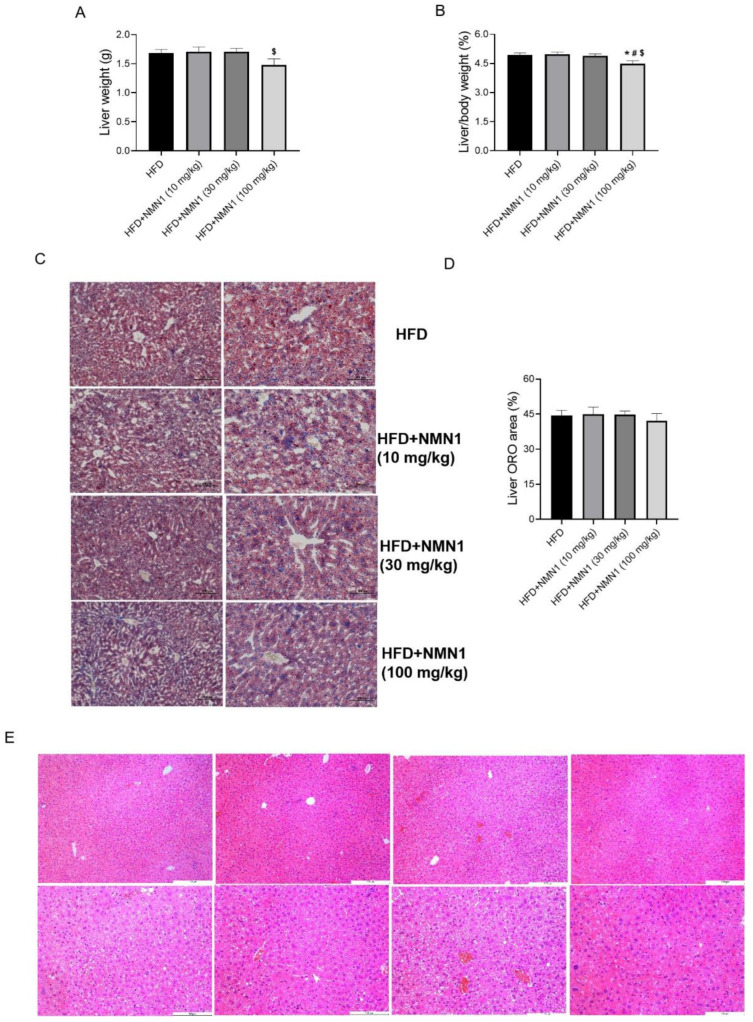
Effects of lower doses of NMN1 (10, 30, 100 mg/kg) on the liver in ApoE^−/−^ mice with HFD for 14 weeks. (**A**) Liver weight (*n* = 11–12), (**B**) liver-to-body-weight ratio (*n* = 11–12), (**C**–**E**) the representative images of Oil red O (**C**) and HE (**E**) staining on the liver and liver ORO area (**D**) (*n* = 11–12) at 14 weeks after treatment. Data represent the mean ± SEM, * *p* < 0.05 vs. HFD group; ^#^ *p* < 0.05 vs. HFD + NMN1 (10 mg/kg) group; ^$^ *p* < 0.05 vs. HFD+NMN1 (30 mg/kg) group. 100× or 200×. NMN: nicotinamide mononucleotide, NR: nicotinamide riboside.

The above different repeated experiments all demonstrated that long-term administration of NMN (300 mg/kg) by gavage or drinking water aggravated atherosclerosis. It is necessary to explore the effect of long-term administration of NMN at reduced doses on atherosclerosis to ensure relative safety. ApoE^−/−^ mice were fed an HFD for 14 weeks, and the three treatment groups were given NMN1 at doses of 10, 30, and 100 mg/kg per day by gavage. Body weight changes were reduced in the 100 mg/kg group at 10 and 14 weeks post-treatment compared to the control group (Figure 9A, *p* < 0.05). There were no significant changes in daily food intake between the four groups during the administration (Figure 9B). Compared with the HFD group, the administration groups did not show any significant alterations in blood glucose levels at 14 weeks (Figure 9C, *p* < 0.05). Mice in the NMN 100 mg/kg group had lower TG and LDL-c levels compared to the HFD control and NMN 10 mg/kg groups. Mice in the NMN 100 mg/kg group had lower TG, TC, and LDL-c levels compared to the NMN 30 mg/kg group, with no other significant changes (Figure 9F). TG/HDL-c and LDL-c/HDL-c ratios decreased only in the NMN 100 mg/kg group (Figure 9E,F). Liver weight decreased slightly only in the NMN 100 mg/kg group (Figure 10A,B, *p* < 0.05). The supplement of NMN at low doses (10, 30, and 100 mg/kg) had no significant effects on liver fat accumulation and hepatic injuries (Figure 10C,D).

**Figure 11:**
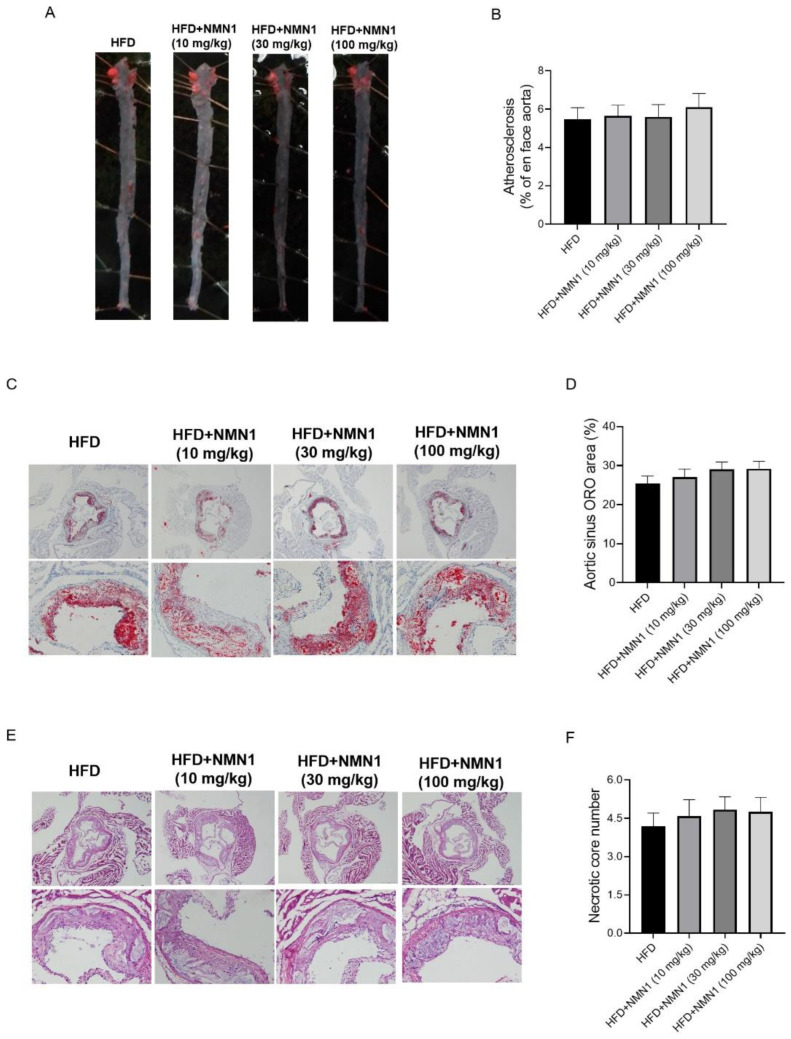
Effects of lower doses of NMN1 (10, 30, 100 mg/kg) on atherosclerosis in ApoE^−/−^ mice with HFD for 14 weeks. (**A**,**B**) The representative images of Oil red O staining on the aorta (**A**) and the statistical results (**B**) (*n* = 11–12), (**C**,**D**) the representative images of Oil red O staining on the aortic root (**C**) and the statistical results (**D**) (*n* = 11–12), (**E**,**F**) the representative images of HE staining on the aortic root (**E**) and the statistical results (**F**) (*n* = 11–12) at 14 weeks after treatment. Data represent the mean ± SEM. 40× or 100×. NMN: nicotinamide mononucleotide, NR: nicotinamide riboside.

Finally, we compared atherosclerosis in the four groups. As shown in Figure 11, although there were no significant differences in the plaque areas on the aorta, lipid accumulations, and necrotic injuries of the aortic root among the four groups, a dose-dependent trend to increased atherosclerosis existed in all three parameters. Thus, unlike high-dose NMN administration, the low doses of 10, 30, and 100 mg/kg had little effect on atherosclerosis.

Altogether, for NMN, both beneficial effects (reducing serum lipids and fatty liver) and harmful effects (promoting atherosclerosis) are dose-dependent and decrease with dose reduction. NMN dose of around 100 mg/kg seems appropriate for beneficial effects with little harmful effects on atherosclerosis.

## 3. Discussion

NMN exists widely in natural foods, including vegetables, fruits, meat, and seafood. After years of development and continuous efforts, the synthesis of NMN has been gradually improved and marketed as nutraceuticals [25]. NMN has a wide range of promising applications in diseases such as obesity, ischemia–reperfusion injury, stroke, aging, and other diseases. The development of drugs with NMN as the active ingredient has become a medical hotspot [12]. In animal experiments, NMN has been demonstrated to have cardiovascular protection mainly through enhancing endothelium-dependent vasodilation and alleviating oxidative stress and inflammatory response [18,19]. Meanwhile, NMN intervention may be a promising strategy for anti-aging, improving glucose metabolism, and even adjuvant treatment of cardiovascular disease in clinical trials. Currently, the majority of clinical trials involving NMN focus more on nutraceutical benefits rather than pharmacological research. In addition, the choice of clinical drug dose and timing of administration needs further discussion [26].

NMN has been shown to reduce serum lipid levels, decrease high-fat diet-induced hepatic lipid deposition, improve glucose tolerance, and enhance insulin sensitivity, which were also related to the development of atherosclerosis [8,27]. NMN supplementation has also been shown to increase energy expenditure activity, thereby attenuating age-related increases in body weight, and long-term administration of NMN for 12 months can reduce age-related weight gain in a dose-dependent manner [27,28]. NR supplementation exerts an anti-obesity effect, prevents inflammation and fibrosis in the white adipose tissue of female mice with diet-induced obesity, and has also been shown to enhance oxidative metabolism, providing protection against obesity [29]. In our study, we confirmed that administration of NMN and NR resulted in weight loss in C57BL/6J mice after one month. However, the impact on weight change in ApoE^−/−^ mice fed a high-fat diet for an extended period of drug administration did not show a clear conclusion, with only occasional significant differences at some time points. This outcome may be attributed to animals of different genotypes, and in-depth research is required for experiments involving drugs from different companies, varying doses, and animals of different ages to better understand the effects. It has been shown that pancreatic beta cells were highly sensitive to decreased levels of NAD+ in the body, and supplementation with NMN significantly enhanced insulin secretion and sensitivity in peripheral tissues, thereby improving glucose tolerance and insulin resistance in aged and diet-induced diabetic mice [8]. However, we did not observe significant direct hypoglycemic effects of NMN and NR in mice. This discrepancy could be due to differences in the timing of blood glucose measurement and administration methods and animal models.

Meanwhile, NMN (300 mg/kg) and NR (230 mg/kg) of different companies were administered by gavage for one month in C57BL/6J mice, and the result showed that they decreased high-fat-induced weight gain and alleviated liver lipid deposition. NMN and NR also significantly reduced LDL-c levels but did not affect TG in mice. This may be due to the atherosclerotic high-fat diet, which itself can reduce the TG level of C57BL/6 mice; therefore, there was no significant difference after administration. The conclusions of the above-mentioned indicators were also consistent for different NMN products made by different companies. Compared with the C57BL/6J mice, NMN and NR showed greater improvement in blood lipid indicators in ApoE^−/−^ mice, which is consistent with the reported results [30]. In addition, the TG/HDL-c ratio serves as a key indicator of metabolic syndrome and cardiovascular disease, exhibiting significant elevation in atherosclerosis [31,32]. Notably, the LDL-c/HDL-c ratio proves more efficient in discerning carotid intima and media thickening, and research underscores the predictive significance of the LDL-c/HDL-c ratio on carotid plaque in postmenopausal females [33,34]. In the present investigation, administration of NMN (300 mg/kg) and NR (230 mg/kg) resulted in a decrease in TG/HDL-c and LDL-c/HDL-c ratio compared to the ApoE^−/−^ mice in the control group, and there was still a significant difference when NMN dose was reduced to 100 mg/kg.

The fact that NMN and NR can improve lipids and fatty liver has been confirmed in many studies, but aggravating atherosclerosis is our first report, and this conclusion has been confirmed with drugs from different companies. However, a distinction arises in that NR2 presents a more severe phenotype of atherosclerosis compared to NMN2, while NR1 does not exhibit such. Further, we also confirmed that the administration (gavage or drinking water) of NMN at 300 mg/kg could still aggravate atherosclerosis in ApoE^−/−^ mice that have developed plaque after 2 months of a high-fat diet. These data strongly suggested that atherosclerosis is aggravated at such a dose. Based on the complexity of the current mechanism of atherosclerosis, we speculate that the results may be due to the following explanations: Firstly, NMN has the potential to lower systemic blood lipid levels while promoting the accumulation of localized fat in the arteries. In the NAMPT-NAD axis, CD38, PARP, and SIRT play important biological roles [5], which are also linked to the pathway of cholesterol efflux and reverse cholesterol transport. Secondly, it has been demonstrated that NMN can activate the NAD+/SIRT1 pathway, thereby reducing inflammatory response and oxidative stress levels in mice, as well as diminishing the expression of COX2 in macrophages [35]. However, recent studies have also revealed that prolonged high-dose NMN supplementation in elderly mice yields positive effects on liver and heart function while increasing the expression of inflammatory markers (IL-1 and CCL2) and tubule injury indicators (Kim-1) in aged kidneys [36]. These findings suggest age and tissue-dependent effects of NMN administration in vivo and dosage considerations for the elderly population. Thirdly, in a recent study exploring molecular mechanisms, SARM1 is a metabolic sensor that induces feedforward metabolic mutations and axonal death by cleaving residual NAD+ in response to an increase in the NMN/NAD+ ratio [37,38]. Extensive evidence has demonstrated that NMN interacts with and stimulates the pro-degenerative enzyme SARM1. Consequently, inadequate conversion of NMN into NAD+ results in the accumulation of toxic NMN, ultimately leading to axonal degeneration [39], and further experiments are needed to determine whether NMN acts through SARM1 in atherosclerosis. In addition, NMN supplementation can surpass the equivalent needs of the human body for NAD+ and may lead to a metabolic burden at high doses. Studies have also confirmed that high doses of NR supplementation deteriorated atherosclerosis and induced systemic inflammation in ApoE^−/−^ mice through increasing hepatic expression levels of CD38 and decreasing SIRT1 [40]. Thus, excessive intake of NMN and NR in the whole body may lead to indirect effects on the vascular system and promote the occurrence of atherosclerosis.

Finally, mice were administered NMN at 300 mg/kg and NR at 230 mg/kg in the study, corresponding to human doses of 24–33 mg/kg and 18–26 mg/kg according to different conversion methods [41,42]. Then, we delved further into the impact of prolonged NMN administration at reduced doses on atherosclerosis, seeking a dose of NMN in mice that exerted no detrimental effects on the condition. At an NMN dosage of 100 mg/kg, the equivalent human dose of 8–11 mg/kg exhibited positive impacts on blood lipids and fatty liver while showcasing no negative effects on atherosclerosis. Meanwhile, doses of 10 and 30 mg/kg were deemed ineffective. In the completed or ongoing clinical trials related to cardiovascular diseases, most participants have been administered doses below 500 mg/d, indicating a relatively safe dose range [10,26]. Recently, a randomized, double-blind clinical trial selected 80 healthy individuals and provided them with NMN at a dose of up to 900 mg/d for a duration of 2 months. Although the results showed a significant increase in circulating NAD+ levels without notable side effects, this underscores the importance of cautious dosage selection in clinical trials [43]. Furthermore, special attention should be paid to elderly patients with arterial plaques, as long-term NMN intake for cardiovascular diseases may potentially deteriorate atherosclerosis. In the future, it is imperative to carry out clinical trials targeting different populations affected by atherosclerosis at varied NMN dosages.

## 4. Materials and Methods

### 4.1. Animals and Groups

**Figure 12:**
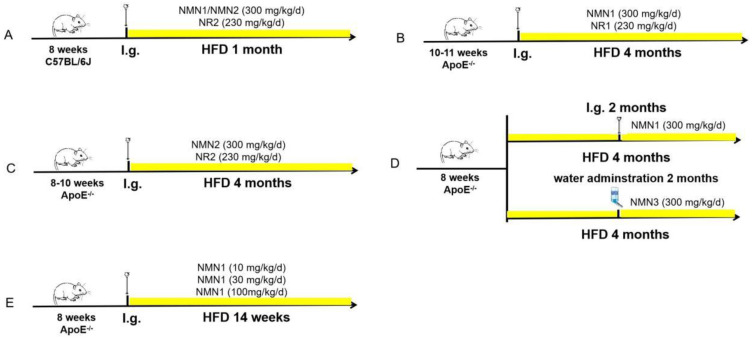
A schematic diagram of experimental design and animal grouping. (**A**) 8-week-old C57BL/6J mice were divided into four groups with HFD for 1 month. (**B**) 10–11-week-old ApoE^−/−^ mice were divided into three groups with HFD for 4 months. (**C**) 8–10-week-old ApoE^−/−^ mice were divided into three groups with HFD for 4 months. (**D**) 8-week-old ApoE^−/−^ mice were divided into four groups with HFD for 4 months. Mice were given NMN1 (300 mg/kg) by gavage in one group and NMN3 (300 mg/kg) by water in another group after 2 months of HFD. (**E**) 8-week-old ApoE^−/−^ mice were divided into four groups with HFD for 14 weeks.

C57BL/6J mice were purchased from Sippe-Bk Lab Animal Co, Ltd. (Shanghai, China). The ApoE knockout (ApoE^−/−^) mice were created by CRISPR/Cas9-mediated genome engineering and purchased from Model Organisms Center, Inc. (Shanghai, China) or Cyagen Biosciences (Suzhou, China). In brief, the ApoE gene was located on mouse chromosome 7, and 4 exons were identified, with the ATG started codon in exon 2 and the TGA stopped codon in exon 4. Exon 2~4 was selected as the target site, and then Cas9 and gRNA were co-injected into fertilized eggs for mouse production. All experimental animals weighed 20–30 g and were randomly divided into different groups as follows (Figure 12). All animals were housed in individual ventilated cage systems with free access to food and water under a 12 h light–dark cycle. Mice were deeply anesthetized intraperitoneally with 1% pentobarbital sodium (Bioszune Life Sciences DEP, Beijing, China) before certain experiments. All animal experiments were performed in accordance with the National Institute of Health Guide for the Care and Use of Laboratory and approved by the ethical committee for animal experiments of the Naval Medical University.

### 4.2. Drugs

**Figure 13:**
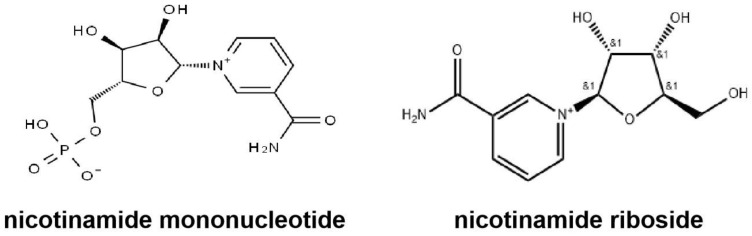
The structural formula of NMN and NR. The left is the structural formula of NMN. The right is the structural formula of NR.

In most of the literature research reports, the dose of NMN used for mouse experiments is typically 300 or 500 mg/kg. We used the dose of 300 mg/kg as the initial dose. Taking into consideration that the molar mass of NMN is 334.2 g/mol and that of NR is 255.3 g/mol, we used an initial dose of 230 mg/kg for NR to ensure equimolarity between the two agents. The chemical composition of NMN1, NMN2, and NMN3 products is equivalent, and the formula is C_11_H_15_N_2_O_8_P. The chemical composition of NR1 and NR2 products is also equivalent, and the formula is C_11_H_15_N_2_O_5_. The structural formula of NMN and NR is shown as follows (Figure 13). Both NMN and NR used in the experiment were supplied by different companies for comparative study. NMN1 was provided by SYNCOZYMES Co., Ltd. (Shanghai, China). NR1 was provided by Natural Field Bio-Technique Co., Ltd. (Xian, China). NMN2 and NR2 were provided by EffePharm Co., Ltd. (Shanghai, China), and NMN3 was provided by Bontac Bio-engineering Co., Ltd. (Shenzhen, China).

### 4.3. Body Weight and Food Intake

ApoE^−/−^ and C57BL/6J mice were fed an atherosclerotic high-fat diet (HFD, Puluteng Biotechnology Co., Ltd., Shanghai, China) containing base feed 60%, lard 16.4%, sucrose 10%, casein 8.5%, cholesterol 1.3%, bile salt 0.3%, premix 1.6%, maltodextrin 1.9% for a period of time. Mice were fed the diet 2–3 times per week, and the weight of the animals was monitored every two weeks. Body weight change = (Final weight-original weight)/original weight × 100%. Food intake was also measured during the administration period, food intake = (initial weight of feed-final weight of feed)/days/number of mice.

### 4.4. Atherosclerotic Lesion Analysis

The aorta was separated layer by layer under a microscope to the common iliac artery of the lower extremities. The heart and kidneys and excess adipose tissue attached to the blood vessels were removed, and the aorta was stained with Oil red O (Sigma-Aldrich, St. Louis, MO, USA). The heart tissue was fixed in 4% paraformaldehyde (Saiweier Biotechnology Co., Ltd., Wuhan, China) for use; then, two steel pins fixed head and tail were taken and began to slowly cut along the longitudinal aorta while cutting the edge to increase the fixation pins, ultimately flattening the entire aortic lumen upwards. The inner surface of the aorta was stained with the Oil red O solution: PBS buffer was dropped, the Oil red O solution was added to the aorta, and the aorta was immersed for 15 min. The Oil red O solution was dropped, and the 75% ethanol (Saiweier Biotechnology Co., Ltd., Wuhan, China) was added to the aorta, and then the aorta was immersed for 10–15 min. The 75% ethanol was dropped, the saline was added, and the image was taken with a digital camera. Image-J software V1.54 (NIH, Bethesda, MD, USA) was used to analyze the lesion area as previously described [23].

### 4.5. Frozen Section and Staining

The tissue and the sample were frozen at −20 °C for about 30 min, and then frozen sections were started. When cutting the target tissue quickly, the thickness of the slice should be adjusted to 10–20 μm, and the slice plane should be observed frequently under the microscope. Each sample was cut into 20 consecutive slices and marked according to the slicing sequence. Each slice was 10 μm thick and placed at room temperature for at least 30 min to prevent detachment, and then frozen at −20 °C. We performed Hematoxylin-eosin staining (HE) staining first. Frozen sections were removed from −20 °C and dried at 37 °C for 1 h. The slices were placed in anhydrous ethanol for 1 min, 95% ethanol for 1 min, 85% ethanol for 1 min, 75% ethanol for 1 min, rinsed under water for 5 min, and hematoxylin for 5 min. Rinse with water for 5 min, 0.5% HCl-75% ethanol for 3 s, rinse with water for 5 min, 75% ethanol for 1 min, eosin for 5 min, anhydrous ethanol for 1 min, blow dry, and seal with neutral gum. Subsequently, oil O red staining was also performed. The frozen sections were taken out from −20 °C and baked at 37 °C for 1 h. The slices were placed in Oil red O dye solution for 15 min, 60% ethanol for 1 min, and ddH_2_O for 1 min; then, the water droplets were removed and dried. Finally, the slices were sealed with glycerin. All of these reagents were purchased from Saiweier Biotechnology Co., Ltd., Wuhan, China. Oil red O and HE staining of the aortic root were performed on all ApoE^−/−^ mice samples taken from each group, and at least 2 slices of each sample were guaranteed to be stained, and 5 different scopes of images from each sample were taken.

### 4.6. Detection of Glucose

Experimental animals were fasted (free to drink) for 12 h (20:00–8:00). Prior to the measurement, the animals were placed in a separate cage and kept free for more than 1 h in a quiet, undisturbed environment. For blood glucose measurement, the end of their tails were cut off by 1–2 mm, and the first drop of blood was gently extruded and wiped clean with a cotton ball. After squeezing out the second drop of blood, blood glucose was measured using a glucose detection instrument (GA-3, Sinocare Inc., Changsha, China).

### 4.7. Serum Lipid Assay

After the mice were completely anesthetized, the thoracic cavity was opened to expose the heart. The blood was slowly extracted from the confluence of the superior and inferior vena cava to the EP tube with a 1 mL syringe. After the blood was placed at room temperature for 2 h, the serum was taken after centrifugation (3000× *g*, 20 min). The levels of triglyceride (TG), low-density lipoprotein cholesterol (LDL-c), high-density lipoprotein cholesterol (HDL-c), and non-esterified fatty acid (NEFA) were detected by an automatic biochemical analyzer. For serum lipid assay, all kits were provided by Nanjing Jiancheng Bioengineering Institute. During the detection process, the components in the blood lipids formed a chemical reaction with the substances in the kit, and the color depth of the red quinone compounds was directly proportional to the content of the blood lipids. The absorbance values of the standard tube and the sample tube were determined, respectively, to calculate the content of the blood lipids.

### 4.8. Measurement of Liver Condition

After the mice were anesthetized and the blood was taken from the heart, the abdominal cavity was opened to expose the liver. The liver tissue was completely excised, and the blood was washed with saline. The excess water was dried with filter paper for weighing. The liver tissue was fixed in 4% paraformaldehyde, and the frozen sections were taken for Oil red O and HE staining as previously described [44]. Oil red O and HE staining of the liver was performed on samples in each group, and at least 2 slices were guaranteed to be stained, and 5 different scopes of images from each sample were taken. The picture was taken by using an upright optical microscope (Leica Microsystems, Wetzlar, Germany) and quantified by the percentage of red area (ORO staining) in the overall area with Image-J software.

### 4.9. Statistical Analysis

Statistical analysis was performed with Gradprism software v12.0. The experimental results were expressed as means ± standard error. In all quantitative data, the comparison of means between two samples was performed using a two-tailed Student’s *t*-test, and the comparison of means among three or more groups was analyzed using one-way ANOVA. A *p*-value less than 0.05 was considered statistically significant.

## 5. Conclusions

In this study, we demonstrated that intragastric intake of NMN (300 mg/kg) and NR (230 mg/kg) led to a reduction in body weight, serum lipids and fatty liver, yet exacerbated atherosclerosis in ApoE^−/−^ mice following 4 months of administration. Atherosclerosis was also deteriorated after 2 months of diverse NMN administration methods in ApoE^−/−^ mice with preexisting plaques. The impact of NMN was contingent on dosage, with doses approximately 100 mg/kg yielding minimal deleterious effects on atherosclerosis. Consequently, NMN and NR ameliorate dyslipidemia and fatty liver but aggravate atherosclerosis in ApoE^−/−^ mice. These revelations underscore the importance of determining a safe dosage for clinical trials involving NMN. Moreover, heightened attention should be given to elderly individuals with arterial plaques, as prolonged consumption of NMN has the potential to exacerbate atherosclerosis.

## Data Availability

All data from the study are included in the manuscript.
